# Evaluation of stresses on mandible bone and prosthetic parts in fixed prosthesis by utilizing CFR-PEEK, PEKK and PEEK frameworks

**DOI:** 10.1038/s41598-023-38288-2

**Published:** 2023-07-17

**Authors:** Yomna H. Shash, Mohamed T. El-Wakad, Mohamed A. A. El-Dosoky, Mohamed M. Dohiem

**Affiliations:** 1 Department of Biomedical Engineering, Helwan University, Cairo, Egypt; 2 Faculty of Engineering and Technology, Future University in Egypt, Cairo, Egypt; 3 Department of Prosthodontics, Zagazig University, Zagazig, Egypt

**Keywords:** Health care, Medical research, Engineering

## Abstract

Fixed prostheses are appropriate treatment solutions for edentulous patients. In fixed prostheses, following “All on four”, titanium frameworks are commonly used to support the implants. However, the limitations of titanium have prompted researchers to search for alternative materials (e.g. polymers). This study applied finite element investigation to evaluate the stress distribution in the parts of fixed prosthesis and the surrounding bone tissue, using polymeric frameworks in place of titanium, and different densities of spongy bone. As, the success of fixed prosthesis was predicted to be influenced also by bone quality, particularly spongy bone density. Fixed prosthesis was constructed on edentulous mandible, then different frameworks (CFR-PEEK 60%, CFR-PEEK 30%, PEKK, and PEEK) were stimulated instead of titanium, under 300N unilateral and bilateral forces. Three densities of spongy bone were stimulated which are normal, low and high. The choice of framework material depended on the density of spongy bone. Moreover, PEEK framework showed the lowest stress values on bone tissues and the highest stress values on mucosa. All frameworks could be used in the fixed prosthesis, in the cases of normal and high densities of spongy bone. In low-density case, soft frameworks (PEKK and PEEK) were recommended to reduce the stresses generated on bone tissues.

## Introduction

Due to the current drawbacks of traditional complete dentures, implant-supported fixed prostheses have recently been employed as appropriate solutions for edentulous patients to restore the quality of life^1^. These prostheses utilize “All-on-4” concept which reduces the treatment time and the risk of morbidity, overcomes the complicated prosthetic and surgical problems caused by anatomical limitations, and also improves eating, speaking, and self-confidence^2,3^. Moreover, the "All-on-4" prostheses are characterized by their high success rates and easy care routines. The drawbacks of "All-on-4" are relatively minor and can be overcome. These drawbacks are presented by their relatively high costs and poor hygiene complications which are rare occurrences^4^.

In “All-on-4”, four implants are placed in the edentulous jaw for supporting the loaded prosthesis, without the requirement for bone augmentation^2–4^. Till now the material of choice for implants is titanium due to its rigidity, strength, excellent corrosion resistance, and biocompatibility with the bone and gum tissues. Titanium, on the other hand, has drawbacks, including aesthetic impairment, metallic taste, and incompatibility with imaging techniques^5^. As a possible alternative to titanium, zirconia implants have been recently used because of their high mechanical stability, biocompatibility, low plaque affinity, and white “tooth-like” colors^6^. However, other researchers have recommended the usage of polymeric implants (e.g. PEEK) because of their elastic modulus which is close to the bone, and their shock-absorbing abilities^7,8^. Despite the drawbacks of titanium, long-term reports about clinical survival rates have made titanium the “gold standard” material over other materials for the manufacturing of endosseous dental implants^9,10^. Histological studies have shown the complete integration of titanium implants with bone tissue, as they can physically bond to bone tissues and hence last longer even if subjected to high stresses, compared to their alternatives^5,9,10^.

In “All-on-4”, a framework is attached to the titanium implants through the multi-unit abutments to support them and enhance the load distribution, reducing the stresses transferred to bone tissues and mucosa. On the framework, acrylic artificial teeth are assembled and secured using acrylic material^3^. In fixed prostheses, one of the key factors for long-term clinical success is the correct choice of the superstructure (framework) material that supports the implants and artificial teeth, and transfers the load to the substructures including the bone tissue^11,12^. Many clinicians want to find suitable framework materials that overcome biomechanical deficiencies while also improving function, aesthetics and the primary stability of the fixed prosthesis. Metallic materials (e.g. titanium) are commonly employed in the manufacturing of frameworks due to their durability, biocompatibility, and great corrosion resistance^5^. However, the patients' desire for using low-cost metal-free prostheses has prompted researchers to look for alternate materials.

PEKK (polyether ketone ketone) and PEEK (polyether ether ketone) are two new polymeric materials that have recently been introduced in dentistry for the manufacture of implants, abutments, and removable and fixed prostheses^7,13^. These materials are biocompatible and radiolucent, with low densities, strong shock absorption abilities, high chemical erosion resistances, and good aesthetic appearances. The mechanical properties of PEEK can be improved to suit the biological demands by adding carbon fibers (CFR-PEEK) in a certain percentage^14^. Carbon-fiber-reinforced polyetheretherketone (CFR-PEEK) composites have historically been used in the production of bone fixation screws, spinal cages, cardiac and neurological leads, and knee replacement products^14,15^. Recently, they have been successfully used in dentistry because of their biocompatibility, and mechanical durability^16^. These composites are expected to be used widely in dentistry in the fabrication of different prosthetic parts, as alternatives to stainless steel and titanium materials. To fabricate a part, CFR-PEEK sheets (30% or 60%) can be heated above their melting temperature and then pulled over or pushed into a mold with either vacuum or pressure^17^. Although polymeric materials have been employed in dentistry and orthopedics, further studies are needed to determine whether they can be used in the fabrication of frameworks.

The primary stability of fixed prosthesis is expected to be influenced by bone quality, as it influences the distribution of loads in all parts. Following edentulism, bone loss is a common occurrence, in which the old bone breaks down quicker than the new one grows, reducing bone density and hence strength. Too much bone production, too little bone resorption, or too much vitamin D in the body, on the other hand, enhances bone density and strength. The researchers also have revealed that the spongy bone is more susceptible to bone loss or gain than the cortical bone. Low and high densities for spongy bone are defined from CT scans as 150 and 850 Hounsfield units [HU]^18,19^.

The finite element analysis has gained popularity in dentistry due to its ability to model complex geometries and provide important information regarding the distributions of stresses/strains in prosthetic parts, surrounding tissue, and bone^20^. In this study, the finite element method was utilized to investigate the effect of using novel polymeric frameworks (e.g. CFR-PEEK 30&60%, PEKK, and PEEK) as alternatives to titanium framework in the fixed prostheses to overcome its limitations. This study also investigated the effect of spongy bone density on the stability of prosthesis and the selection of framework material. 300 N unilateral and bilateral forces were applied vertically to stimulate the different mastication mechanisms. The null hypothesis predicted that the polymeric frameworks would exhibit the lowest stresses in bone tissues and represent the best scenario, in comparison to the titanium frameworks. Additionally, these polymeric frameworks would be appropriate for use with both high and low densities of spongy bone, not only in the normal density case.

## Results

### Stresses on framework

Tables 1 and 2 illustrated the max von Mises stresses on all frameworks, in all cases, under unilateral and bilateral forces. On the titanium framework, under unilateral force (Table 1), the max stress was 102.2 MPa in the normal-density case. This value was increased by 11.25% on CFR-PEEK 60% framework and decreased by 57.49, 79.15, and 83.73% on CFR-PEEK 30%, PEKK, and PEEK frameworks respectively. In the high-density case, the max stress was increased by 11.22% on CFR-PEEK 60% framework, and decreased by 56.78, 78.72 and 83.36% on CFR-PEEK 30%, PEKK and PEEK respectively. The same happened in the low-density case, as the stress on CFR-PEEK60% framework was increased by 11.53%, in comparison to titanium, while the stresses on CFR-PEEK 30%, PEKK, and PEEK were decreased by 59.7, 80.38 and 84.74% respectively.

**Table 1:** Max von Mises stresses (MPa)on framework, implants and mucosa, under unilateral force.

	Framework	Implants	Mucosa
Normal density			
TI	102.2	63.13	0.071
CFR-PEEK 60%	113.7	63.48	0.068
CFR-PEEK 30%	43.45	62.64	0.096
PEKK	21.31	61.51	0.115
PEEK	16.63	60.99	0.121
High density			
TI	99.71	61.36	0.059
CFR-PEEK 60%	110.9	62.24	0.056
CFR-PEEK 30%	43.09	59.49	0.084
PEKK	21.22	57.88	0.104
PEEK	16.59	57.25	0.110
Low density			
TI	109.3	53.53	0.097
CFR-PEEK 60%	121.9	53.53	0.094
CFR-PEEK 30%	44.05	52.78	0.120
PEKK	21.45	51.81	0.138
PEEK	16.68	51.40	0.145

**Table 2:** Max von Mises stresses (MPa)on framework, implants and mucosa, under bilateral force.

	Framework	Implants	Mucosa
Normal density			
TI	104.2	64.01	0.086
CFR-PEEK 60%	116.7	64.62	0.083
CFR-PEEK 30%	44.99	63.00	0.111
PEKK	22.12	62.24	0.129
PEEK	17.53	61.59	0.135
High density			
TI	101.9	61.62	0.074
CFR-PEEK 60%	114.1	61.98	0.070
CFR-PEEK 30%	44.36	60.53	0.100
PEKK	21.92	58.73	0.119
PEEK	17.41	57.98	0.126
Low density			
TI	109.8	56.46	0.110
CFR-PEEK 60%	122.9	56.65	0.106
CFR-PEEK 30%	45.92	55.14	0.132
PEKK	22.36	53.96	0.149
PEEK	17.66	53.48	0.155

Under bilateral forces (Table 2), the max von mises stresses on titanium frameworks were 104.2, 101.9 and 109.8 MPa in normal, high, and low densities cases respectively. These values were increased to 116.7, 114.1, and 122.9 MPa on CFR-PEEK 60% frameworks, however, decreased to 17.53, 17.41, and 17.66 MPa on PEEK frameworks.

### Stresses on implants

For the three cases, the highest stress values on implants were found by utilizing CFR-PEEK 60% frameworks, as shown in Tables 1 and 2. In comparison to the titanium framework, CFR-PEEK 30%, PEKK, and PEEK frameworks resulted in reductions in the max stresses of implants. These reductions were (0.78, 2.57 and 3.39%) in the normal-density case, (3.05, 5.67 and 6.7%) in the high-density case, and (1.4, 3.21 and 3.98%) in the low-density case, under unilateral force.

Under bilateral force, by using CFR-PEEK 30%, PEKK, and PEEK frameworks, the reductions in the stresses of implants were (1.56, 2.76 and 3.78%) in the-normal density case, (1.76,4.69 and 5.9%) in the high-density case and (2.33, 4.42 and 5.27%) in the low-density case, in comparison to titanium framework.

### Stresses on mucosa

Tables 1 and 2 demonstrated that the PEEK frameworks increased the mucosal stresses, compared to other frameworks. Moreover, under unilateral force, the highest stress value (0.145 MPa) was found in the low-density case, followed by (0.121 MPa) in the normal-density case and (0.11 MPa) in the high-density case. In addition, CFR-PEEK 60% decreased the mucosal stress to (0.071 MPa), (0.059 MPa), and (0.097 MPa) in normal, high, and low densities cases respectively.

Under bilateral force, by using titanium frameworks, the max stresses on mucosa were (0.086 MPa), (0.074 MPa), and (0.11 MPa) in normal, high, and low densities cases. These values were increased to (0.135 MPa), (0.126 MPa) and (0.155 MPa) by using PEEK frameworks, however, reduced to (0.083 MPa), (0.07 MPa) and (0.106 MPa) by using CFR-PEEK60% frameworks.

**Figure 1:**
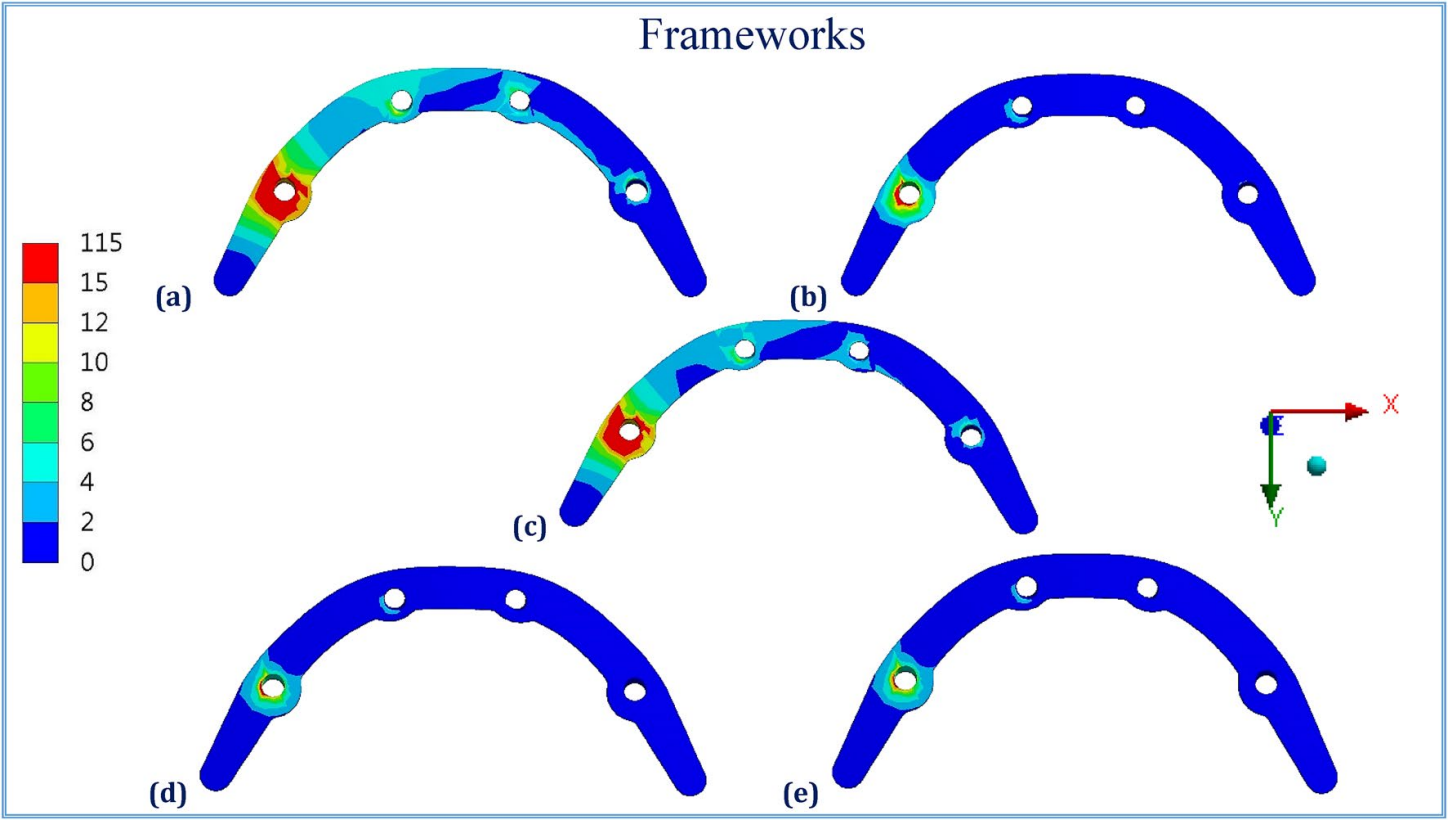
The distribution of von Mises stresses (MPa) on the frameworks, in the normal-density case, under unilateral force. (**a**) CFR-PEEK 60%, (**b**) CFR-PEEK 30%, (**c**) Titanium, (**d**) PEKK, (e) PEEK.

**Figure 2:**
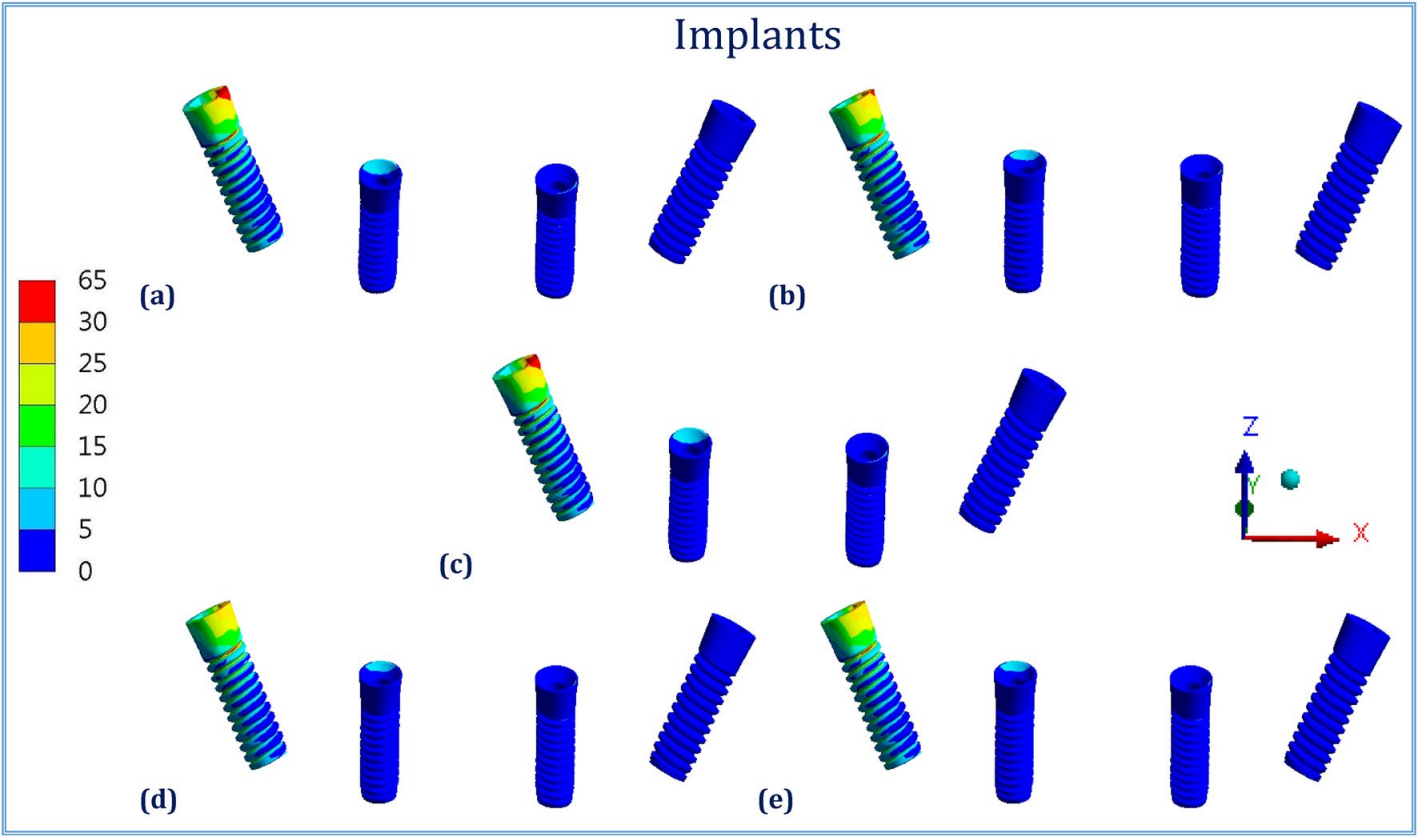
The distribution of von Mises stresses (MPa) on the implants, in the normal-density case, under unilateral force. (**a**) CFR-PEEK 60%, (**b**) CFR-PEEK 30%, (**c**) Titanium, (**d**) PEKK, (**e**) PEEK.

**Figure 3:**
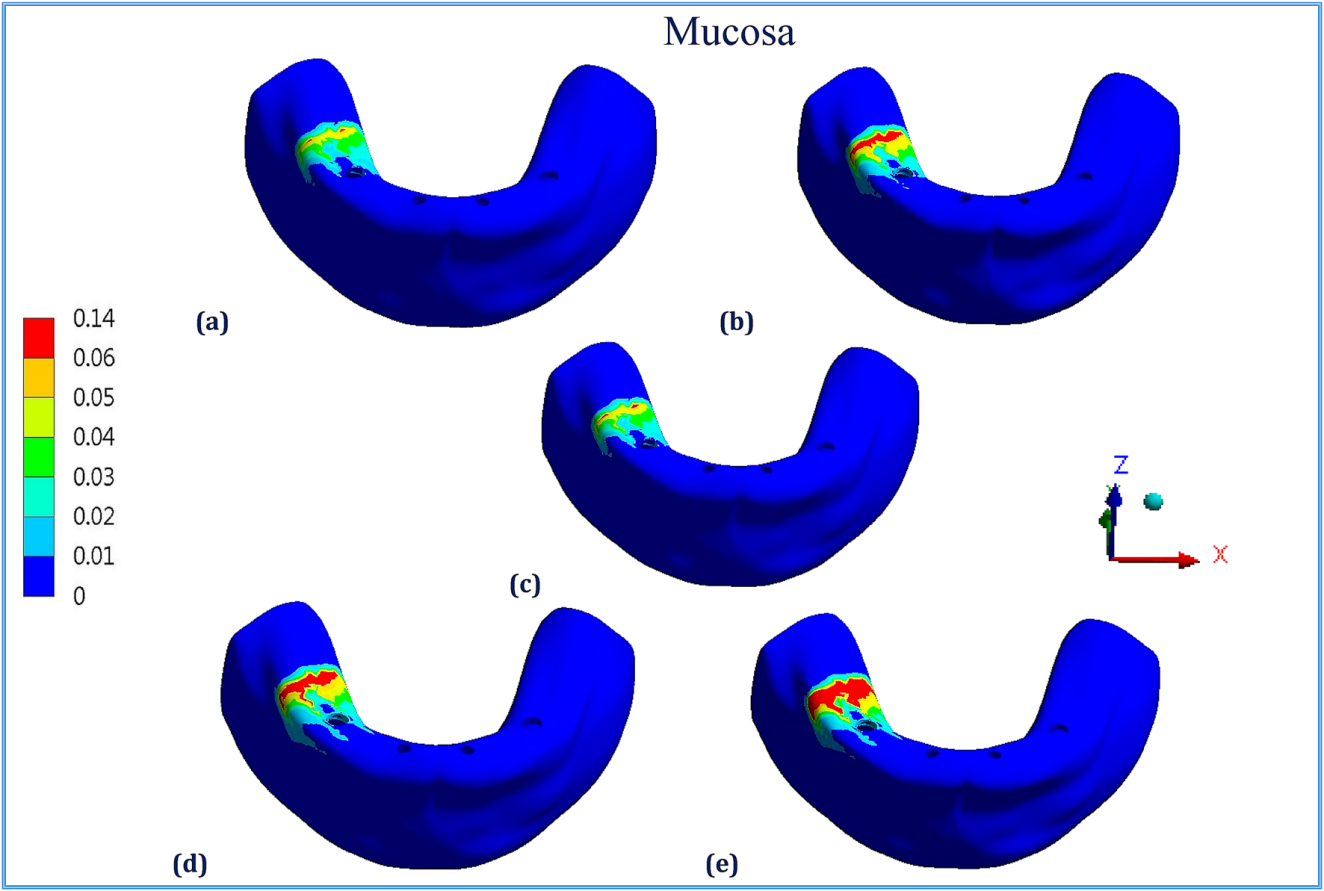
The distribution of von Mises stresses (MPa) on the mucosa, in the normal-density case, under unilateral force. (**a**) CFR-PEEK 60%, (**b**) CFR-PEEK 30%, (**c**) Titanium, (**d**) PEKK, (**e**) PEEK.

**Figure 4:**
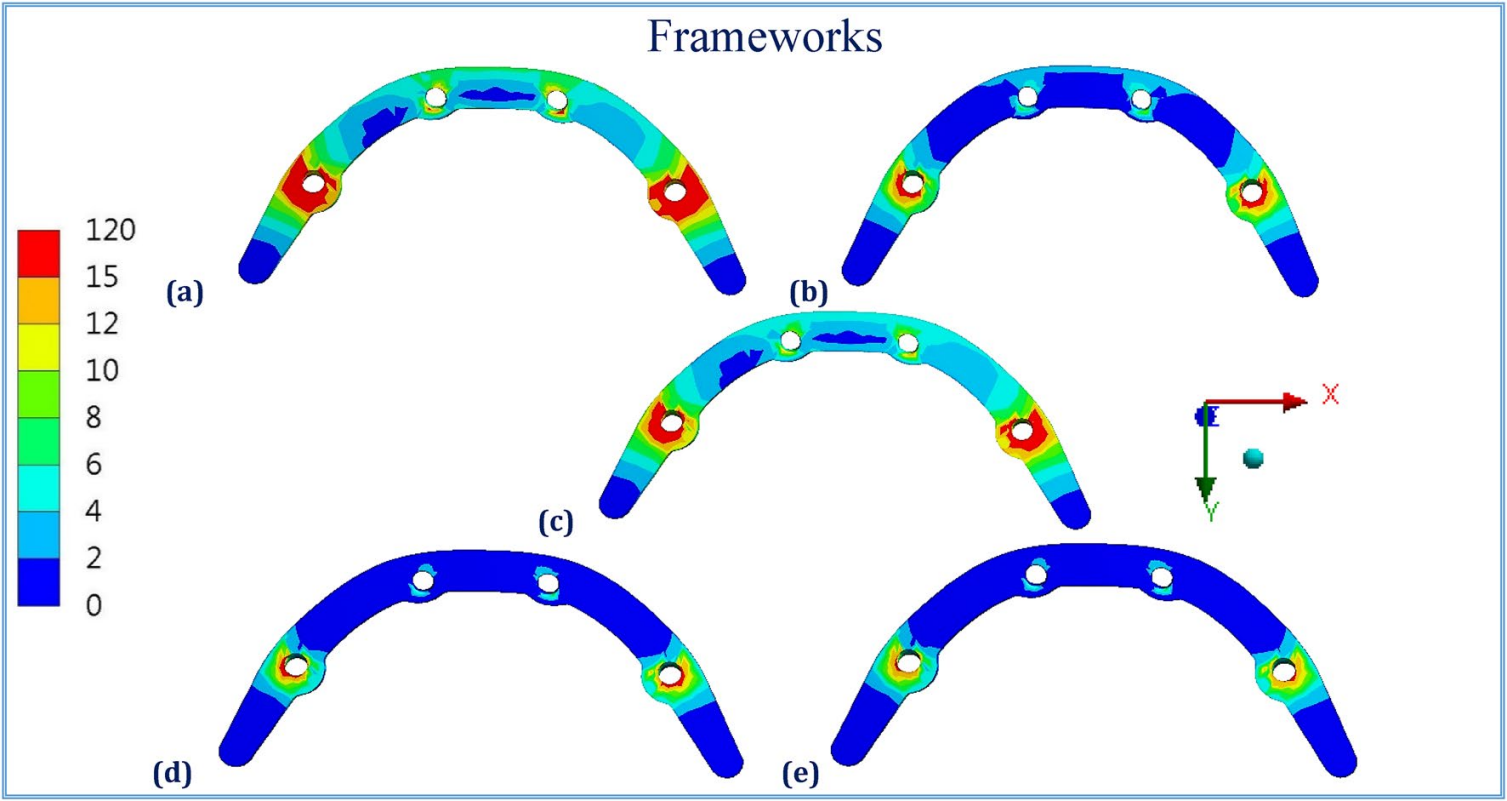
The distribution of von Mises stresses (MPa) on the frameworks, in the normal-density case, under bilateral force. (**a**) CFR-PEEK 60%, (**b**) CFR-PEEK 30%, (**c**) Titanium, (**d**) PEKK, (**e**) PEEK.

**Figure 5:**
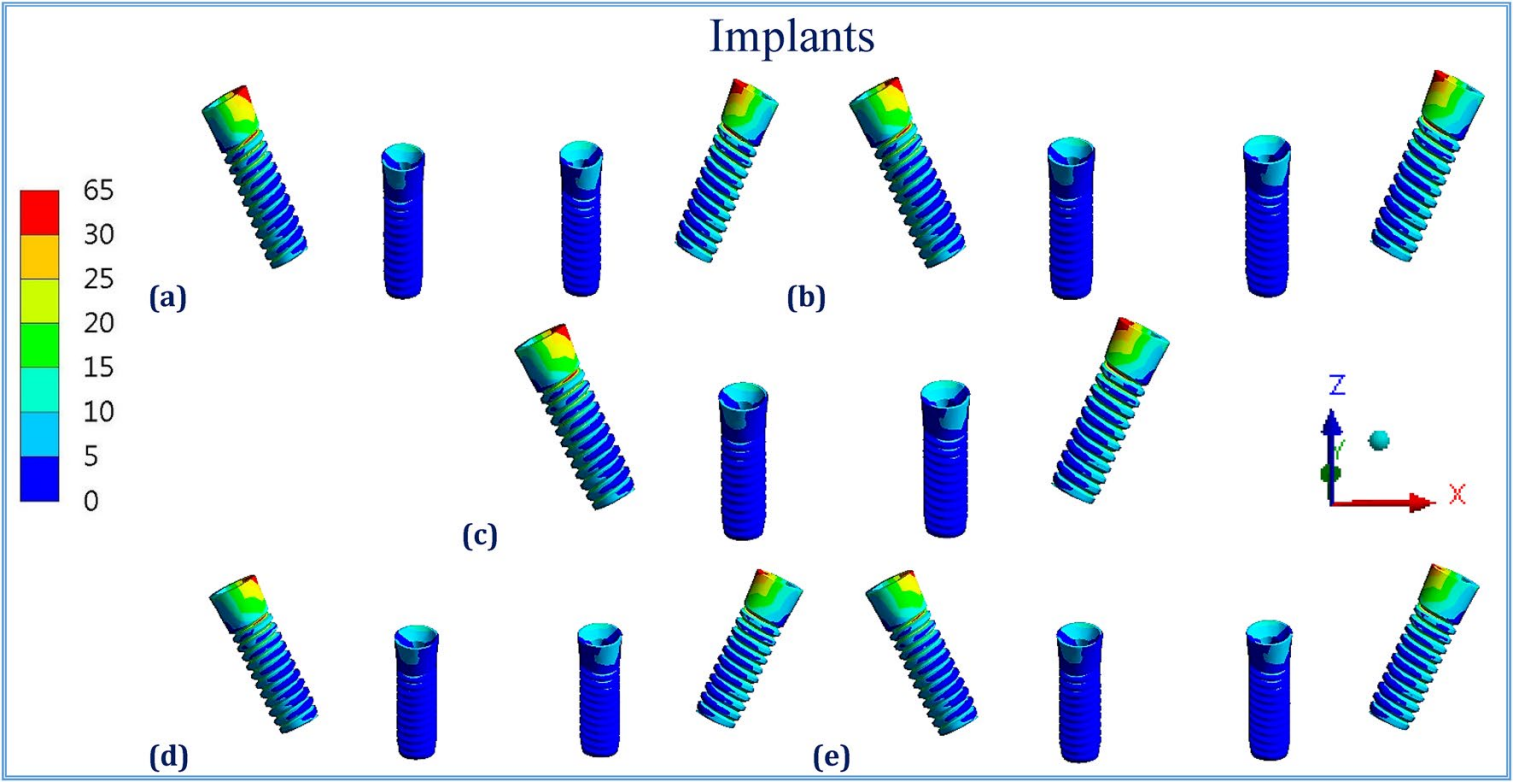
The distribution of von Mises stresses (MPa) on the implants, in the normal-density case, under bilateral force. (**a**) CFR-PEEK 60%, (**b**) CFR-PEEK 30%, (**c**) Titanium, (**d**) PEKK, (**e**) PEEK.

**Figure 6:**
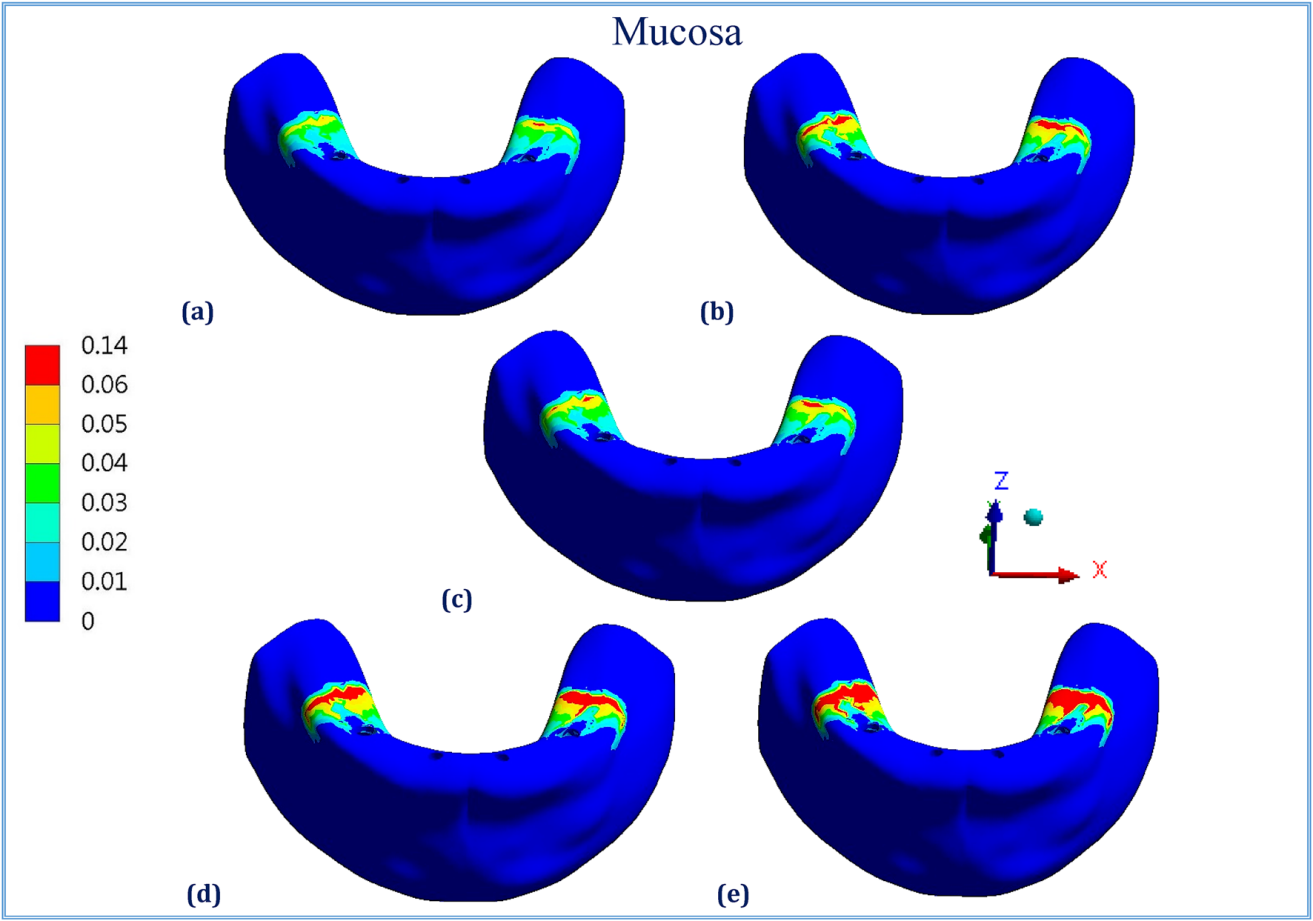
The distribution of von Mises stresses (MPa) on the mucosa, in the normal-density case, under bilateral force. (**a**) CFR-PEEK 60%, (**b**) CFR-PEEK 30%, (**c**) Titanium, (**d**) PEKK, (**e**) PEEK.

Figures 1, 2, 3, 4, 5 and 6 illustrated the distribution of von Mises stresses (MPa) on frameworks, implants and mucosa, by using CFR-PEEK 60%, CFR-PEEK 30%, titanium, PEKK and PEEK frameworks, in normal density case of spongy bone, under unilateral and bilateral forces.

### Stresses on cortical and spongy bones

For all three cases, under unilateral and bilateral forces, the lowest values of peak max and min principal stresses of cortical bone were recorded in the high-density case, followed by the normal-density case and the low-density case (Tables 3, 4). In addition, CFR-PEEK 60% framework similarly distributed the stresses as the titanium framework.

**Table 3:** Maximum and minimum principal stresses (MPa) on cortical and spongy bones, under unilateral force.

	Cortical bone		Spongy bone	
	Max	Min	Max	Min
Normal density				
TI	18.05	− 44.24	2.75	− 4.23
CFR-PEEK 60%	18.06	− 44.48	2.76	− 4.23
CFR-PEEK 30%	17.87	− 43.60	2.63	− 4.19
PEKK	17.76	− 42.67	2.57	− 4.12
PEEK	17.67	− 42.00	2.56	− 4.10
High density				
TI	8.75	− 35.33	2.92	− 4.75
CFR-PEEK 60%	8.77	− 35.73	2.94	− 4.75
CFR-PEEK 30%	8.61	− 34.14	2.90	− 4.69
PEKK	8.54	− 33.55	2.86	− 4.61
PEEK	8.49	− 33.30	2.85	− 4.58
Low density				
TI	40.14	− 73.26	1.84	− 2.194
CFR-PEEK 60%	40.18	− 73.29	1.85	− 2.197
CFR-PEEK 30%	39.98	− 73.06	1.80	− 2.178
PEKK	39.81	− 72.90	1.75	− 2.15
PEEK	39.66	− 72.76	1.74	− 2.14

**Table 4:** Maximum and minimum principal stresses (MPa) on cortical and spongy bones, under bilateral force.

	Cortical bone		Spongy bone	
	Max	Min	Max	Min
Normal density				
TI	16.79	− 52.27	2.93	− 4.235
CFR-PEEK 60%	16.84	− 52.98	2.96	− 4.25
CFR-PEEK 30%	16.33	− 48.45	2.78	− 4.21
PEKK	16.03	− 46.27	2.67	− 4.14
PEEK	15.70	− 45.65	2.63	− 4.11
High density				
TI	9.89	− 43.17	3.00	− 4.71
CFR-PEEK 60%	9.93	− 43.80	3.03	− 4.73
CFR-PEEK 30%	9.64	− 39.86	2.84	− 4.68
PEKK	9.53	− 37.99	2.77	− 4.61
PEEK	9.51	− 37.47	2.76	− 4.58
Low density				
TI	37.36	− 71.11	1.84	− 2.143
CFR-PEEK 60%	37.42	− 71.91	1.85	− 2.145
CFR-PEEK 30%	36.56	− 66.68	1.79	− 2.134
PEKK	36.01	− 64.58	1.75	− 2.104
PEEK	35.52	− 64.58	1.72	− 2.090

Under unilateral force, in the normal-density case, the max principal stress on cortical bone was reduced by 1, 1.61 and 2.11% by using CFR-PEEK 30%, PEKK, and PEEK frameworks, compared to titanium. Besides, the min principal stress was reduced by 1.45, 3.55, and 5.06% respectively. In the high-density case, for CFR-PEEK 30%, PEKK, and PEEK frameworks, the value of max principal stress was reduced by 1.6, 2.4, and 2.97%, compared to titanium. In addition, the value of min principal stress was reduced by 3.37, 5.04, and 5.57%. In the low-density case, the values of max and min principal stresses for cortical bone were nearly unchanged. For spongy bone, using CFR-PEEK 30%, PEKK, and PEEK instead of titanium, the max principal stress was reduced by (4.36, 6.55 and 6.91%) in the normal-density case, (0.68, 2.05 and 2.39%) in the high-density case, and (2.17, 4.89 and 5.43%) in the low-density case, as illustrated in Table 3. Moreover, the min principal stress was reduced by (0.95, 2.6 and 3.07%) in the normal-density case, (1.26, 2.95 and 3.57%) in the high-density case, and (0.73, 2.01 and 2.46%) in the low-density case respectively.

Under bilateral force (Table 4), in the normal-density case, the max principal stress on cortical bone was reduced by 2.73, 4.52 and 6.49% by using CFR-PEEK 30%, PEKK, and PEEK frameworks, compared to titanium. Moreover, the min principal stress was reduced by 7.3, 11.47, and 12.66% respectively. For CFR-PEEK 30%, PEKK, and PEEK frameworks, in the high-density case, the value of max principal stress was reduced by 2.52, 3.64 and 3.84%, and the value of min principal stress was reduced by 7.66, 11.99 and 13.20%. In the low-density case, the value of max principal stress on cortical bone was reduced by 2.14, 3.61 and 4.92% for CFR-PEEK 30%, PEKK, and PEEK frameworks, and the value of min principal stress was reduced by 6.22, 9.18 and 9.18% respectively, compared to titanium framework. For spongy bone, using CFR-PEEK 30%, PEKK, and PEEK instead of titanium, the max principal stress was reduced by (5.11, 8.87 and 10.23%) in the normal-density case, (5.33, 7.66 and 8%) in the high-density case, and (2.71, 4.89 and 6.52%) in the low-density case as illustrated in Table 4. Moreover, the min principal stress was reduced by (0.59, 2.24 and 2.95%) in the normal-density case, (0.63, 2.12 and 2.76%) in the high-density case, and (0.41, 1.81 and 2.47%) in the low-density case respectively.

**Figure 7:**
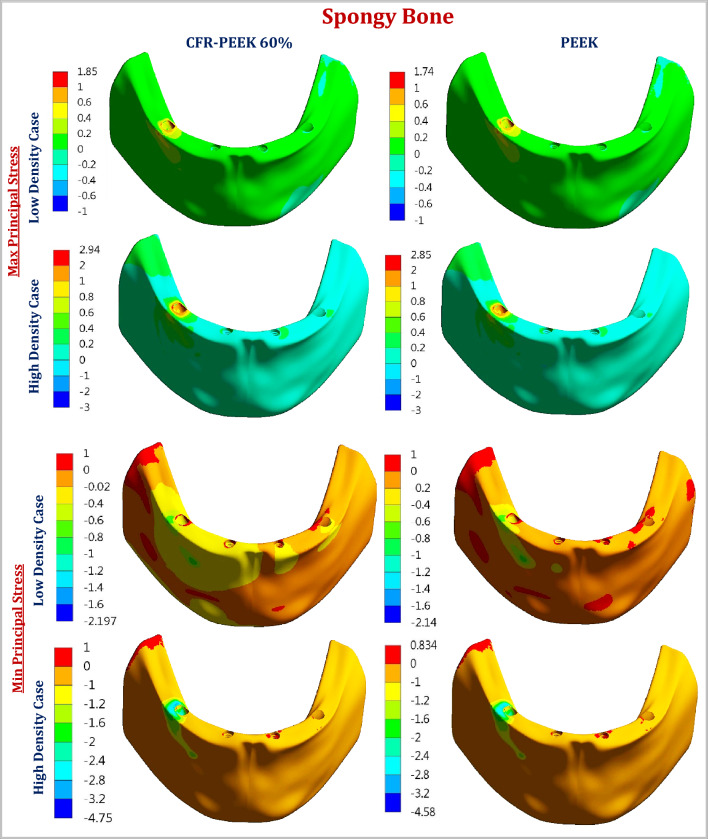
The distribution of max and min principal stresses (MPa) on spongy bone by using CFR-PEEK 60% & PEEK frameworks, under unilateral force. In the low-density case & the high-density case.

**Figure 8:**
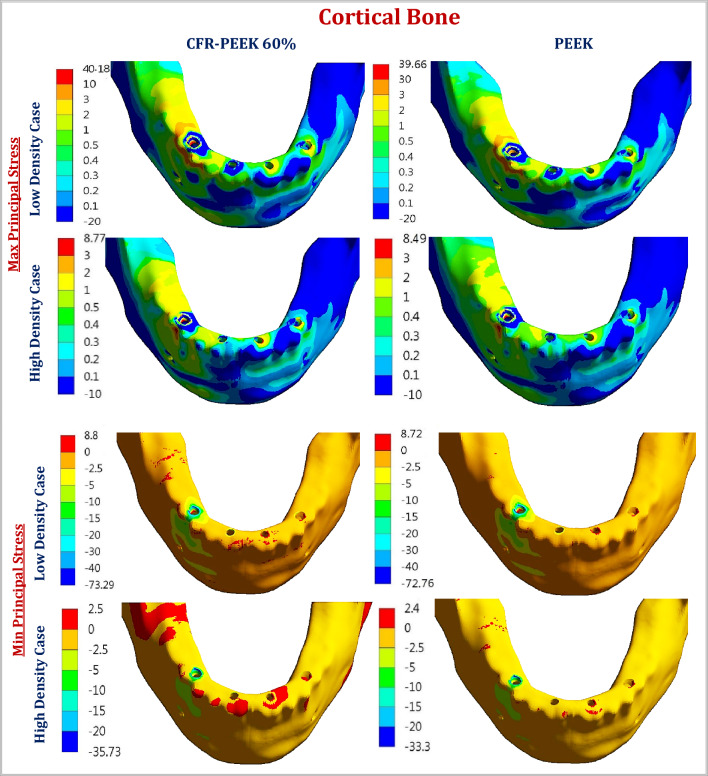
The distribution of max and min principal stresses (MPa) on cortical bone by using CFR-PEEK 60% & PEEK frameworks, under unilateral force. In the low-density case & the high-density case.

**Figure 9:**
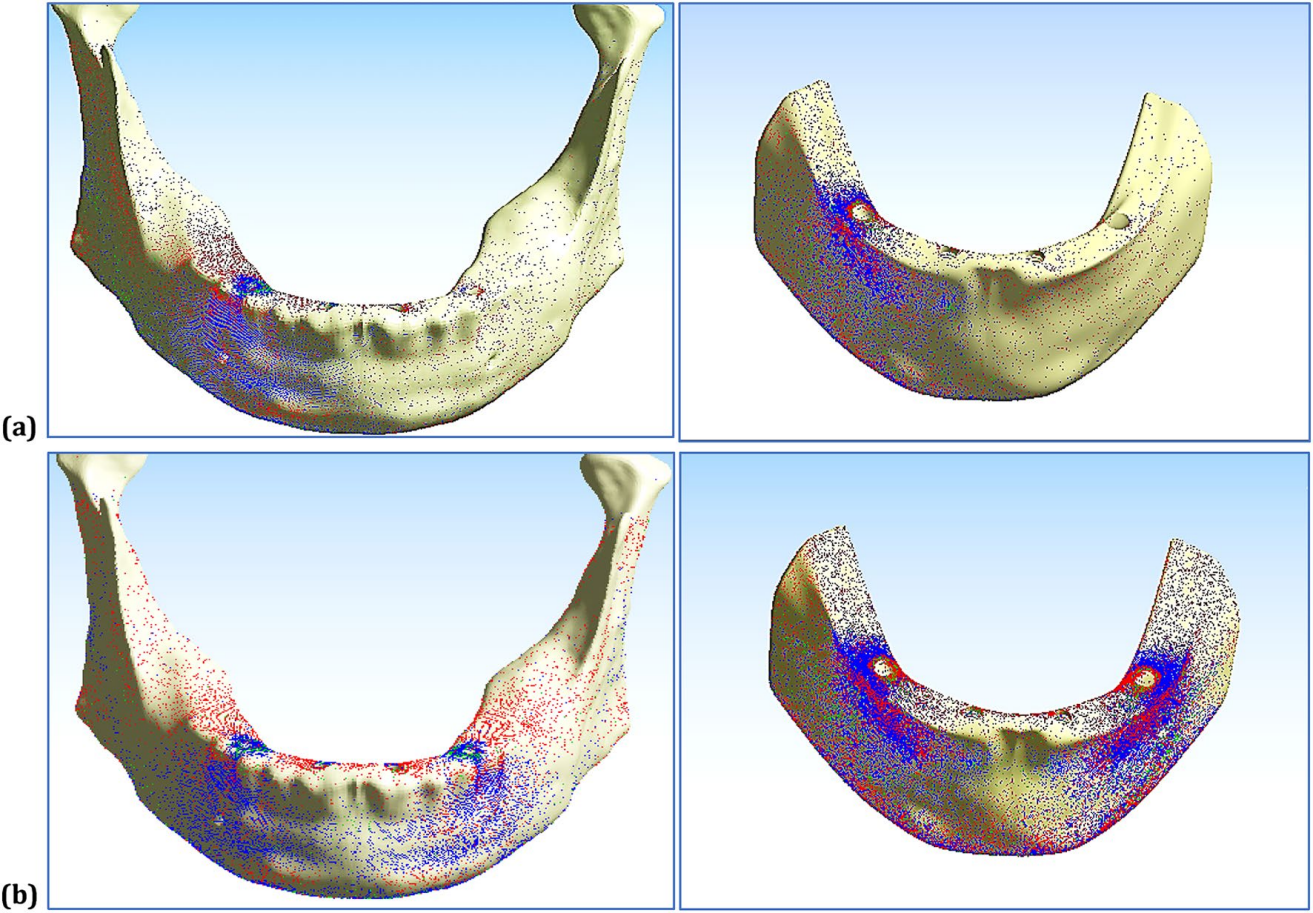
Principal stress vectors for cortical and spongy bones, in the normal-density case, under: (**a**) Unilateral force, (**b**) Bilateral force.

Figures 7 and 8 illustrated the distribution of max and min principal stresses (MPa) on cortical and spongy bones by using CFR-PEEK 60% &PEEK frameworks, in the low and high densities cases, under unilateral force. Figure 9 illustrated the principal stress vectors, under unilateral and bilateral forces for cortical and spongy bones. The red and blue arrows indicated the areas with tension and compression respectively. For cortical bone, most stresses were compressive around the loaded holes, while both compressive and tensile stresses were distributed in the spongy bone around these holes.

### Implant displacement

**Figure 10:**
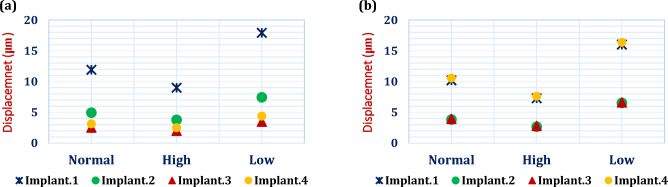
The max values of implants displacements, under: (**a**) Unilateral force, (**b**) Bilateral force.

Using the three cases of cancellous bone density, the maximum total displacement values (μm) of the four implants were retrieved and compared with the critical limits (50–150 μm^21^) to assess the primary stability of the fixed prosthesis. Under unilateral force, implant 1 (loaded side), as seen in Fig. 10, displayed the largest displacement values, while implants 3 & 4 (non-loaded side), displayed the lowest values. Under bilateral force, implants 1&4 displayed the largest displacement values than other implants. In addition, the four implants also showed larger displacements in the low density case than in the other cases.

## Discussion

The stress–strain analysis is a branch of engineering that utilizes different techniques to determine the stresses and strains in materials and structures, under different loading conditions. The photoelastic technique, digital image correlation technique, electrical resistance strain gauge, and finite element method have been commonly employed in dentistry in the stress–strain analysis^20–24^. The finite element method (FEM) is a numerical method of analyzing the stresses and strains at any point. It has several advantages over other techniques in dentistry, including the ability to accurately represent complex geometries, make modifications, suggest new designs, specify multiple boundary conditions, stimulate various materials, and then quickly calculate the internal stresses and strains at any point^20^.

This study used the finite element method to conduct stress analysis on edentulism mandible rehabilitated with a fixed prosthesis, using different frameworks. The study focused on the new polymeric frameworks (CFR-PEEK 30&60%, PEKK and PEEK) to be used as alternatives to the protocol titanium frameworks, besides revealing their effects on the biomechanical performance of the fixed prosthesis. Different densities of spongy bone were stimulated to choose the appropriate framework material for each bone condition. In Shash et. al's research^25^, a 3D model of a mandible with a fixed prosthesis was constructed and stimulated with different frameworks, using only the normal case of bone density. The simulation results demonstrated that the polymeric frameworks could be used instead of titanium in the fabrication of the hybrid prostheses.

According to the von Mises Yield Criterion^26^ and studies^27–30^, the maximum von Mises stresses were calculated for prosthetic parts (frameworks and implants) because of their ductile properties. In the mandible, the cortical bone is much stiffer and stronger than the spongy bone, while the spongy bone is more flexible and ductile. According to the failure theory of principal stress^26^ and like other studies^28,31,32^, the maximum (tensile) and minimum (compressive) principal stresses were extracted for cortical and spongy bones and compared to the permissible tensile and compressive strengths (Table 5), due to their brittle and ductile properties. To investigate the yielding or failure behavior of each bone tissue. To stimulate the mechanism of mastication, 300N vertical force was applied unilaterally and bilaterally on the three posterior teeth. Hence, high stresses were concentrated at the elements beneath the applied force, in all models as FEA studies^19,27–32^.

**Table 5:** The mechanical properties of all parts included in study.

	Elastic Modulus (GPa)	Poisson Ratio	Yield Strength (MPa)
Mucosa	0.005	0.4	
Cortical bone	13.7	0.3	*
Spongy bone			
Normal density	1.37	0.3	**
Low density (150HU)	0.259	0.3	***
High density (850HU)	3.5	0.3	****
PMMA	5	0.37	
Titanium	110	0.35	825
CFR-PEEK 60%	150	0.42	2100
CFR-PEEK 30%	18	0.4	300
PEKK	5.1	0.4	200
PEEK	3.5	0.37	150

*Tensile strength = 100 MPa & Compressive strength = 140 MPa.

**Tensile strength = 10 MPa & Compressive strength = 16 MPa.

***Tensile strength = 3 MPa & Compressive strength = 3.5 MPa.

****Tensile strength = 15 MPa & Compressive strength = 22.5 MPa.

The framework material influenced the stresses generated on all prosthetic parts and bone tissues in studies^12,28,33^, whereas another study found no significant effect^34^. Because of their endurance and superior properties, some authors advised utilizing stiff materials (metals and ceramics) in the fabrication of frameworks. They hypothesized that stiff superstructures would transfer fewer stresses to the substructure parts^5,35,36^. Other authors^7,13,37^ recommended utilizing soft superstructures to dampen the stresses conveyed to the substructure parts.

PEKK and PEEK are polymeric materials, which have recently been used in dentistry in the fabrication of implants, abutments, fixation screws, etc. as alternatives to metals and ceramics^7,13^. These materials are biocompatible in vivo and in vitro and radiolucent, with good mechanical, chemical, thermal, and electrical properties (e.g. resistance to high temperature, hydrolysis, and corrosion), and good aesthetic appearance. Besides, they have neither toxic effects nor clinical inflammation. Due to their structures, low elastic modulus (3–5 GPa) and high shock-absorbing abilities, these materials are expected to solve the stress-based problems on bone tissues. As they can evenly distribute the stresses generated during mastication on the substructure parts and hence reduce the stresses transferred to bone tissue^7,13,27,28^.

Polyether ketone ketone (PEKK) is a novel polymeric material that has attracted the attention of researchers because of its excellent properties that make it suitable for a variety of uses (both industrial and military)^13^. PEKK has recently been employed in restorative, prosthetic, and implant dentistry, because of its superior mechanical strength, shock absorbing ability, low density, and the presence of the second ketone group which enables more surface modification. PEKK material has been introduced as a promising alternative material for long-term orthopedic applications over titanium. Since it offers excellent biocompatibility, and metal-free restorations and is thus helpful in patients with allergies^13^.

PEEK material is a high-performance polymer that has been tested in many medical fields, under different conditions. The tests demonstrated that PEEK material possessed a higher strength-to-weight ratio which is especially important for dental parts. It can also handle abrasive and compressive forces without losing its integrity and durability^7,8^. Moreover, it is lighter than metal and more comfortable for patients. The short-term report^38^ clarified that PEEK-resin prosthesis utilizing "All-on-4" concept, was a viable option for edentulous patients, although they still need long-term evaluations. In addition, the use of PEEK as a framework for a fixed partial denture (FPD) produced very good results with a high level of patient comfort and acceptability, according to Sinha et al.'s case study^39^. Hence, clinically, PEEK material is a premium innovative prosthetic solution for either fixed prostheses, removable or screwed-in.

In finite element investigations, Haroun et al.^28^ extracted the max and min principal stresses generated on normal bone density, using PEEK and titanium frameworks, on the maxillary prosthesis utilizing "All-on-4". The results demonstrated that PEEK material produced the lowest stresses on the bone under unilateral force. The max principal stresses on cortical bone were 23.2 and 34.3 MPa by using PEEK and titanium, while the min principal stresses were 39.5 and 68 MPa respectively. In the current study, in the normal-density case, the values of max principal stresses on cortical bone were 17.67 and 18.05 MPa using PEEK and titanium respectively, while the min principal stresses were 42 and 44.24 MPa. Chen et al.^40^ also conducted finite element analyses of the mechanical performance of four removable partial denture designs, utilizing cobalt chrome, titanium alloy, and PEEK frameworks. The findings demonstrated that the PEEK framework caused the lowest stress on the framework, the lowest stress on the periodontal ligament, and the maximum mucosal stress when compared to cobalt chrome and titanium frameworks.

PEEK properties can be improved to be used in dentistry by the addition of carbon fibers^14^. The elastic modulus of PEEK is about 3.5 GPa, and the addition of carbon fibers with a certain percentage increases the elastic modulus to 18–150 GPa. These composites have recently been used in dentistry due to their strength, resistance to chemical erosion, compatibility with imaging techniques, and high inertness^14–16^. There have been several studies that have evaluated the performance of CFR-PEEK material in a variety of orthopedic implants, under different testing techniques. Most articles have reported positive findings regarding the properties, technical outcomes, durability, and safety of CFR-PEEK in orthopedic implants^14–16,41,42^.

The choice of framework material was also expected to depend on the bone quality to maintain the prosthesis stability. Some studies found a correlation between bone density and prosthesis stability^18,19,43,44^, while others^45,46^ have shown no significant correlations. Marquezan et al.^43^ and Hsu et al.^44^ demonstrated that spongy bone density played an essential influence on prosthetic stability.

In this study, the null hypothesis—assuming that the polymeric frameworks would exhibit the lowest stresses in bone tissues and represent the best scenario, in comparison to the titanium frameworks—was partially accepted. The results illustrated that CFR-PEEK 30%, PEKK and PEEK frameworks reduced the stresses on implants and bone tissues, however, increased the mucosal stress, in comparison to titanium framework, unlike CFR-PEEK 60%. The results illustrated also that the spongy bone density affected the stresses generated on all parts especially mucosa, and bone tissues, for all the used frameworks. In the low-density case, the decrease in spongy bone density decreased the spongy bone stress and thus increased the stresses on cortical bone and mucosa, while the opposite occurred in the high-density case. From the extraction of results, no failure might occur in all frameworks and implants as their max stresses did not surpass their yield strengths (Table 5). The results also clarified that all utilized frameworks (excluding CFR-PEEK 60%) increased the values of mucosal stress, but these values did not exceed the pain threshold value (0.63 MPa^47^), indicating no occurrence of mucosal inflammation.

The maximum and minimum principal stresses were extracted for bone tissues, and compared with the tensile and compressive strengths, according to the failure theory of principal stress^26^. For cortical bone, with normal density, the allowable tensile and compressive limits are (66.6 & 93.3 MPa), using a 1.5 safety factor. For spongy bone, the allowable tensile and compressive limits are nearly (2 &2.33 Mpa), (6.5 &10.5 Mpa), and (10 &15 Mpa) for low, normal, and high densities^48,49^. Tables 3 and 4 illustrated that the maximum and minimum principal stresses of cortical bone did not surpass the allowable limits in all cases. In the normal and high densities cases, the maximum and minimum principal stresses for spongy bones were far from the limits. In the low-density case, the stresses approached the limits, therefore soft frameworks (PEEK & PEKK) were preferred.

The displacements of implants were also extracted in this study using all frameworks and compared to the threshold (50–150 μm^21^) in order to evaluate the primary stability of the prosthesis. Due to the possibility of fibrous tissue forming between the implant and the bone, displacements higher than 150 μm must be avoided^21^. All of the implants in this investigation showed displacements that were less than 50 μm, demonstrating the prosthesis stability.

The assumption that the materials were isotropic and linearly elastic was one of the study's limitations. Additionally, the study was carried out using static loads. It was important to illustrate these limitations in discussing the findings, as the oral tissues and the bone are more intricate and anisotropic structures, besides the mastication mechanism is more complex. Moreover, the implants were assumed to achieve complete osseointegration with bone and this may not accurately reflect the clinical scenario. Also, the posterior implants in this study were tilted at an angle of 30 degrees in accordance with the "All-on-4" configuration technique, however, changing the angle may produce different results. Additionally, altering the type, number, or location of implants may alter all the findings. Consequently, the finite element analysis may not accurately mimic the actual clinical situations.

## Conclusion

Within the limitations of the study, the following were concluded:

- Soft frameworks (PEEK & PEKK) slightly reduced the stresses on bone tissues, however, increased the mucosal stress.
- CFR-PEEK 60% distributed the stresses in a similar manner as the titanium framework.
- All frameworks could be used as alternatives to titanium in normal and high densities cases.
- In the low-density case, soft materials were preferred as they decreased the stresses generated on bone tissues.

In vivo, polymeric frameworks are anticipated to offer numerous major benefits over titanium, including enhanced performance and aesthetics, improved design freedom, production of lighter prostheses, reduced overall cost, and reduced production problems and the risk of mechanical problems. However, additional investigations and long-term studies are needed in near future.

## Methods

### Model geometry

**Figure 11:**
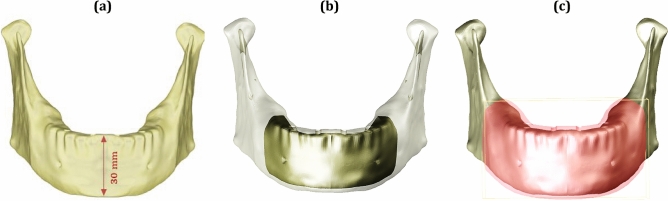
(**a**) Mandible, (**b**) Cortical and spongy bones, (**c**) Mucosa.

A 3D model of a female edentulous mandible was built using an OBJ file imported from "BodyParts3D/Anatomography" website (BodyParts3D, © Life Sciences Integrated Database Center, Japan)^50^. The mandible model was imported as a solid cortical bone with a symphysis height of 30 mm, as shown in Fig. 11a. Utilizing Solidworks software (Version 21, Massachusetts, USA), the mandible was segmented into a cortical bone with 2 mm thickness and a remaining inner volume from the spongy bone (Fig. 11b), with a mandibular ramus being predominantly cortical. Besides, the mandible was covered with 2 mm mucosa as shown in Fig. 11c.

Following “All‑on‑4” concept, anteriorly, vertical implants were modeled with a diameter of 3.7 mm and a length of 10 mm, in the lateral incisal area based on the dimensions imported from ZIMMER catalog (Biomet Dental, Palm Beach Gardens, USA^51^). Posteriorly, two implants were modeled with a diameter of 4.1 mm and a length of 11.5 mm and placed with an angle of 30 into the second premolar region. Anterior implants were fitted with straight multi-unit abutments which did not engage the internal hex connections, while posterior implants were fitted with angled multi-unit abutments (ZIMMER, Biomet Dental, Palm Beach Gardens, USA). Four titanium copings (sleeves) were mounted over the anterior and posterior abutments using mini screws with 1.25 mm diameters.

**Figure 12:**
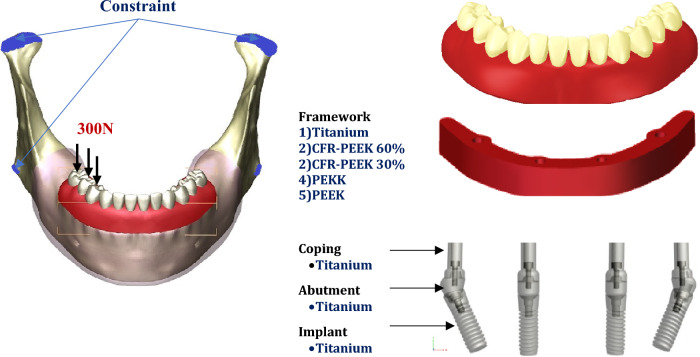
Finite element model & unilateral load and constrains.

A horseshoe-shaped framework (with 5.5 mm height, 5 mm width, and 10 mm cantilever length) was attached to the four copings. Then twelve acrylic teeth were arranged on the framework and secured with acrylic material (Fig. 12). The fixed prosthesis had fewer posterior teeth than the complete denture as the cantilever extension must not go beyond the first molars^12^. The final prosthesis had a height of 15 mm from the surface of the mucosa and a cantilever length of 15 mm (1.5 anterior–posterior spread^52^) as presented in Fig. 12. The acrylic part in the prosthesis covered a small part of the mucosa to absorb some of the mechanical stress instead of bone and prevent the accumulation of food in the mucosa-prosthesis space^53^. Complete osseointegration between implant and bone surfaces was assumed, and all parts were assumed to be perfectly bonded together through the contact surfaces.

### Material properties

In order to save the calculation times, bone tissues were represented with isotropic properties, especially when a complex model was used^54^. The materials used in this study (Table 5) were considered to be homogeneous, linearly elastic, and isotropic. The materials used for the framework were titanium as control, polymers (PEKK &PEEK), and PEEK composites containing 30& 60% carbon fibers. The denture base and artificial teeth were from acrylic PMMA, while the implants, abutments and copings were from titanium^5,7,13,14,19,48,49^.

### Meshing

**Figure 13:**
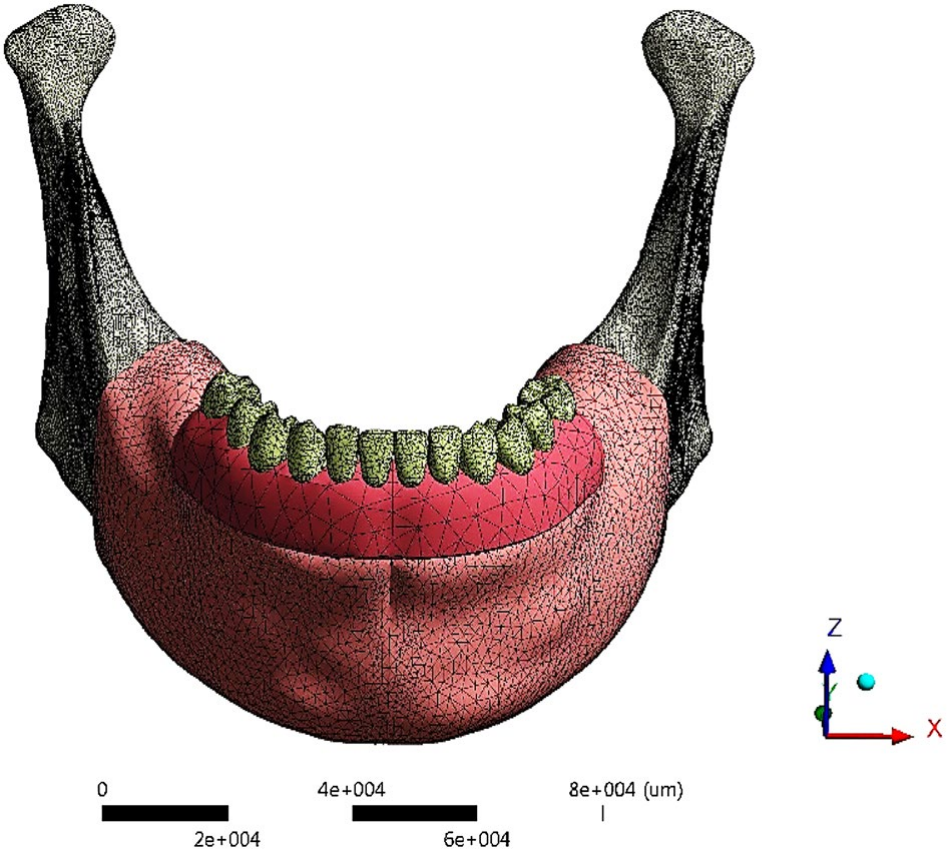
Meshing.

3D meshing was generated in ANSYS software (Version 18.0, Canonsburg, USA), using “adaptive” function with (0.2–1 mm) elements sizes. A mesh refinement was established based on the 10% convergence test. The numbers of nodes and elements in each part were presented in Table 6. The final model (Fig. 13) had approximately 434,577 elements and 598,453 nodes.

**Table 6:** The number of nodes &elements at each part.

	No. of elements	No. of nodes
Mucosa	102,366	18,048
Cortical Bone	201,405	352,551
Spongy Bone	29,283	52,049
Denture Base & Teeth	32,058	57,103
Framework	1679	3241
Copings	3990	7348
Screws	8970	15,906
Abutments	12,521	22,198
Implants	42,305	70,009

### Load and constrains

In fixed prostheses, the average force was around 200–300N in the premolars and molars region^30,55^. In this research, 300N vertical force was applied to the three posterior teeth to stimulate unilateral (Fig. 12) and bilateral mastication mechanisms. To prevent the displacement and rotation of the model during force application, the nodes of condyles and the inferior border of the mandible were restrained in all directions^29,30^.

### Stress analysis

The maximum principal stress (Pmax) and minimum principal stress (Pmin) were computed for bone tissues due to their ductile and brittle properties. Prosthetic components, implants and mucosa were considered as ductile materials and hence their von Mises stresses were calculated. To check the primary stability of the fixed prosthesis, the total displacement of implants was investigated.

## Data Availability

The datasets generated during and/or analyzed during the current study are available from the corresponding author on reasonable request.
